# Intergenerational burden: deconstructing the dual impact of familial suicide in a specialized bipolar clinic, from clinical risk to internalized stigma

**DOI:** 10.3389/fpsyt.2026.1931254

**Published:** 2026-10-20

**Authors:** Fatma Seher Kocaayan, Ezgi Şişman, Havva Atasoy Kaplan, İlay Dalkıran, Hanife Yılmaz Abaylı, Aslıhan Polat

**Affiliations:** 1 Department of Psychiatry, Kocaeli University Research and Application Hospital, Kocaeli, Türkiye; 2 Department of Psychiatry, Kocaeli City Hospital, Kocaeli, Türkiye; 3 Department of Psychiatry, Derince Training and Research Hospital, Kocaeli, Türkiye; 4 Department of Psychiatry, Eyüpsultan State Hospital, İstanbul, Türkiye

**Keywords:** bipolar disorder, family history of completed suicide, internalized stigma, mood disorder, resilience

## Abstract

**Background:**

This study aimed to assess the level of internalized stigma in individuals with bipolar disorder (BD) during the euthymic phase and to examine its individual, familial, and clinical predictors.

**Method:**

Ninety-seven euthymic participants with BD, diagnosed according to DSM-5 criteria, were administered the Internalized Stigma of Mental Illness Scale (ISMI), Resilience Scale for Adults (RSA), and Rosenberg Self-Esteem Scale (RSES).

**Results:**

High levels of internalized stigma (ISMI mean ≥ 2.5) were found in 13.4% of participants. Multiple linear regression analysis showed that higher resilience (β = –0.39, p <.001) and higher educational attainment (β = –0.18, p = .029) were associated with lower internalized stigma, whereas a family history of death by suicide was associated with higher stigma (β = 0.20, p = .019). A greater number of mood episodes showed a borderline association with higher stigma (β = 0.18, p = .050). The overall model explained 52% of the variance in stigma scores.

**Limitations:**

The cross-sectional design and relatively small sample size are the primary limitations.

**Conclusions:**

Internalized stigma in BD was associated with a combination of individual, familial, and educational factors. The independent association of a family history of suicide underscores the importance of a systematic evaluation of such a family history in a clinical interview, suggesting that strengthening resilience and health literacy may represent vital targets for intervention.

## 1 Introduction

Bipolar disorder (BD) is a chronic and disabling mental illness characterized by episodes of depression and mania or hypomania (1). BD, besides the acute episodes that profoundly disrupt life, has an enduring impact during the euthymic phase when many continue to suffer from pronounced psychosocial difficulties, low functioning, and a poor quality of life despite symptom remission (2). One of the main causes behind these ongoing struggles is defined as internalized stigma, where people with mental illness internalize social prejudice and turn that on themselves (3). Internalized stigma is expressed through a range of maladaptive cognitive (e.g., feelings of inferiority), emotional (e.g., shame, hopelessness), and behavioral (e.g., social withdrawal, illness concealment) reactions (4). As a result of this process, they may start to see themselves as “defective” or “inferior”, which can adversely affect their mental health, relationships, treatment adherence and future goals (5).

Research on stigma in BD, although highly relevant, has until recently been less extensively investigated than that of schizophrenia (6, 7). The unequal attention given to BD in research is concerning given that individuals’ experience level of internalized stigma is said to be equal to or even greater than that of schizophrenia (7–9). In fact, many individuals with BD report that they experience stigma in society as well as within their family and social networks (10). Investigations have also demonstrated that revealing a BD diagnosis leads to social distance and social rejection from peers who view them as dangerous (11). These findings highlight an important gap in the literature and call for more nuanced research on the mechanisms and correlates of stigma in the BD population. The gap shows that BD-specific aspects would require exploring. An episodic but chronic condition may be associated with different stigma experiences compared to those associated with persistent conditions (e.g. schizophrenia).

The effects and repercussions of internalized stigma are as significant as the stigma experienced publicly. It has been a major obstacle to achieving life goals by debilitating self-esteem and self-efficacy (12, 13), thereby damaging social position, relationships and occupational functioning (4). Most critically, stigma is a significant barrier to clinical recovery. Many people postpone seeking psychiatric care or prematurely end their treatment because they fear being labelled (6). Within this context, treatment adherence is pivotal in addressing avoidance behaviors, as its disruption often exacerbates the prognosis of BD (12, 14). The experience of internalized stigma varies and seems to be determined by a number of factors. Lower educational attainment and living in rural areas were associated with higher stigma levels (15, 16). Contemporary research suggests that resilience, self-compassion and self-esteem could mitigate the effects of internalized stigma (17, 18). However, less is known about whether familial clinical context, particularly a family history of death by suicide, contributes to internalized stigma when considered together with these established sociodemographic, clinical, and psychological correlates in euthymic individuals with BD.

Drawing upon these gaps in the literature, the study intends to systematically examine the correlates of internalized stigma in BD in the euthymic period. The goal of this paper is to (i) evaluate the prevalence and intensity of internalized stigma in this population, (ii) identify the key sociodemographic, clinical and psychological factors associated with stigma and (iii) study family history of suicide as a relatively less studied variable due to its probable sociocultural importance and stigmatizing potential in our country.

In sociocultural contexts in which mental illness is experienced not only at the individual level but also within the family system, stigma may extend to relatives through processes of associative or familial stigma (19). Families may consequently experience social judgment, concealment, or social distancing related to a relative’s psychiatric illness (19). This burden may be particularly relevant following a death by suicide, as suicide-bereaved family members have been reported to experience shame, blame, social judgment, avoidance, and secrecy (20). Therefore, among individuals with bipolar disorder, a family history of death by suicide may represent an important but underexplored source of stigma-related vulnerability. Based on the existing literature, we hypothesized that higher psychological resilience and higher self-esteem would be associated with lower internalized stigma. Conversely, indicators of greater illness burden, particularly a higher number of previous mood episodes, and familial vulnerability, particularly a family history of death by suicide, were expected to be associated with higher internalized stigma.

## 2 Materials and methods

### 2.1 Study design, participants and procedure

This descriptive, cross-sectional study consisted of outpatients with bipolar disorder who were under regular follow-up at a specialized mood disorders unit of a university-affiliated tertiary care psychiatry department. This clinic provides treatment by pharmacotherapy, psychotherapy and psychosocial interventions to adopt a comprehensive biopsychosocial approach. Patients and their caregivers are routinely given psychoeducation. Medical follow-up includes laboratory tests and psychological assessments. Additionally, the specialized units engage in residency training, scientific research and prevention of stigma associated with mood disorders.

The study protocol was approved by the institutional Non-Interventional Clinical Research Ethics Committee (Approval Date: 25.09.2025, Decision No: 2025/503 KÜ GOKAEK-2025/19/28). The study was performed following the principles laid down in the Declaration of Helsinki. All participants gave written informed consent prior to their involvement in the research.

To participate in the study, individuals had to meet the following requirements: an established diagnosis of BD according to the Diagnostic and Statistical Manual of Mental Disorders, Fifth Edition (21); between 18 and 70 years of age; and maintained clinical stability (euthymia) for six months, which was defined as continuous outpatient follow-up without change in medication regimen. The participants were fully informed about the study procedures, risks and benefits and written informed consent was taken from all. On the assessment day, specialist psychiatrists verified euthymia as defined by Young Mania Rating Scale (YMRS) and Hamilton Depression Rating Scale (HAM-D) scores of ≤7.

Data were collected in a single visit: clinicians completed a case report form; participants self-administered the Rosenberg Self-Esteem Scale (RSES), the Resilience Scale for Adults (RSA), and the Internalized Stigma of Mental Illness Scale (ISMI); and prior clinical history (outpatient visits and hospitalizations) was confirmed via hospitals’ electronic registries. The total number of mood episodes was determined by the treating psychiatrist through a structured face-to-face interview and cross-referenced with the hospital’s electronic medical records. Major depressive, manic, and mixed episodes meeting DSM-5 criteria were recorded as separate episode subtypes when supported by clinical records or sufficient clinical documentation. Hypomanic episodes were not quantified separately because their lifetime frequency was based mainly on unverified self-report and could not be consistently confirmed from clinical records. Episodes that could neither be confirmed through clinical documentation nor judged clinically reliable based on the interview and available records were not counted. Because episode history was assessed retrospectively, the total number of episodes should be interpreted as an approximate indicator of lifetime illness burden rather than a precise or fully verifiable lifetime count. A brief medical screening was conducted to rule out neurological or systemic diseases that could affect neural or cerebrovascular function. Exclusion criteria were the presence of manic, hypomanic, or major depressive symptoms at interview; cognitive impairment; dementia; intellectual disability; DSM-5 alcohol or substance use disorder; severe physical illness that precludes participation; and any comorbid psychiatric disorder. To ensure diagnostic clarity, personality disorders were screened using the Structured Clinical Interview for DSM-5 Personality Disorders (SCID-5-PD), and individuals meeting criteria for any additional DSM-5 psychiatric diagnosis were excluded. All sociodemographic and clinical characteristics were recorded by the researcher during face-to-face interviews using a structured information form.

### 2.2 Sample size

We carried out an *a priori* power analysis in G*Power (version 3.1.9.7) to determine the sample size for the multiple linear regression analysis. Following a medium effect size (f² = 0.15), significance level (α) of.05, desired statistical power (1−β) of.95 and including a model with 15 predictor variables.

The analysis revealed that a minimum of 89 participants was required. The decision to recruit 100 participants was made because of possible loss or incomplete data. Initially, a total of 103 people were recruited for the study. Accordingly, six participants were excluded for failing to complete the questionnaire; therefore, the final sample included 97 which was sufficient for the analyses planned.

### 2.3 Data collection ınstruments

#### 2.3.1 Sociodemographic and clinical information form

This form, developed by the researcher, was administered through face-to-face interviews and completed by the physician. It included questions on participants’ age, gender, marital status, educational level, lifestyle, place of residence, occupation, employment status, age at illness onset, total number of hospitalizations, family history of psychiatric disorders and suicide, history of suicide attempts, comorbid medical conditions, as well as tobacco, alcohol, and substance use.

#### 2.3.2 Hamilton Depression Rating Scale

The HDRS, which was developed in 1960 by Max Hamilton is able to measure the severity of depression. The original version of the scale is administered by a clinician that assesses the patient’s depressive symptom status during the past week. Inclusion of 17 items (22). It measures the intensity of depressed mood suicidal thoughts, reduction of work and activities, retardation and agitation, anxiety, genital and gastrointestinal symptoms, general somatic symptoms, hypochondriasis, insight and insomnia (22). The total score ranges from 0 to 53. A score between 0–7 shows no depression; 8–15 shows mild depression; 16–28 shows moderate depression; and 29 or above shows severe depression. A study was conducted in Türkiye by Akdemir et al. in 1996 to investigate the validity and reliability of the scale (23).

#### 2.3.3 Young Mania Rating Scale

The Young Mania Rating Scale, a measure of the severity of manic symptoms, was developed by Young et al. in 1978 (24). The clinician fills it out based on the interview and behavior observations made during the session (24). The original version of the scale, which considers symptoms occurring in 48 hours, contains 11 items that evaluate 5 primary symptom domains (24). Seven items are scored on a scale of up to 4 points, while the remaining four items are scored on a scale of up to 8 points. The domains assessed include mood, increased motor activity and energy, sexual interest, sleep, irritability, speech (rate and amount), thought disorder, content of thought, disruptive–aggressive behavior, appearance, and insight (24). The Turkish validity and reliability study of the scale was conducted by Karadağ et al. in 2001 (25).

#### 2.3.4 Resilience Scale for Adults

Friborg et al. (2003) created the Resilience Scale for Adults in order to identify protective factors that work in the individual, the family and in external support systems to help people cope in a functional way and prevent mental illness (26). The original version of the scale consisted of 37 items and assessed five dimensions: personal competence, structured style, social competence, family cohesion, and social resources (26, 27). In 2005, to provide a better conceptual explanation of resilience, the personal competence dimension was divided into perception of self and perception of future, resulting in the current 33-item, six-dimension structure (27).

The scale uses a semantic differential format, in which positive and negative attributes are presented on opposite ends, and responses are marked on a five-point scale to minimize biased evaluations (28). There is no cut-off score; higher scores indicate greater levels of psychological resilience. The Turkish validity and reliability study of the scale was conducted by Basım et al. in 2011 (28).

#### 2.3.5 Rosenberg Self-Esteem Scale

The Rosenberg Self-Esteem Scale (RSES), developed by Rosenberg, is a widely used self-report instrument for assessing global self-esteem (29). In the present study, the Turkish version adapted and validated by Çuhadaroğlu was used (30). This version uses a scoring procedure based on Guttman scaling, yielding a total score ranging from 0 to 6. In this scoring system, higher scores indicate lower self-esteem; scores of 0–1 are interpreted as high self-esteem, 2–4 as moderate self-esteem, and 5–6 as low self-esteem. Therefore, in all analyses and interpretations in the present study, higher RSES scores represent lower self-esteem. Because this Guttman-based scoring system differs from the more commonly used Likert-summed versions of the RSES, direct comparison of absolute scores across studies using different scoring methods should be made with caution.

#### 2.3.6 Internalized Stigma of Mental Illness Scale

The Internalized Stigma of Mental Illness Scale was developed by Ritsher et al. in 2003 and this self-report instrument assesses the subjective experience and level of stigma in individuals with mental illness and through the public’s understanding which helps in the conceptualization of stigma (31). The tool has 29 items under five subscales social withdrawal, alienation, presumed discrimination, support from the members of the stigmatized community and stigma tolerance. Each item was assessed using a four-point Likert-type scale (31). The reverse coding of the stigma resistance subscale also acts as a validity check. Higher scores indicate more severe internalized stigma (2, 5, 31).

In some studies, mean item scores ranging from 1 to 4 are calculated with a cut-of point defined at 2.5 (2, 5, 32). Thus, a mean score of 2.5 or higher indicates that an individual has moderate-to-high internalized stigma, whereas mean scores below 2.5 indicate low internalized stigma (2, 5, 32–36). The scale’s Turkish validity and reliability study was carried out by Ersoy et al. in 2007 (37).

### 2.4 .Statistical analysis

The data was analyzed by IBM SPSS Statistics (Version 25). The statistical significance threshold value was determined as *p* <0.05. Numerical data with normal distribution were presented with mean and standard deviation (*SD*) values, and skewed distribution was presented with median and interquartile range (IQR) values. Kolmogorov-Smirnov test was used to test normality. Normally distributed numerical data were analyzed with Student’s *t*-test and Pearson’s correlation. Numerical data that failed the normality tests were assessed with the Mann-Whitney *U* test and Spearman correlation. Categorical data were analyzed with Chi-square and one-way analysis of variance tests.

The statistical analysis was conducted in two stages. First, for preliminary bivariate analyses, participants were categorized based on their Internalized Stigma of Mental Illness Scale (ISMI) mean item score. Participants with a score < 2.50 were assigned to a “low stigma” group, while those with a score ≥ 2.50 were assigned to a “moderate–high stigma.

For continuous variables, between-group comparisons were performed using Student’s independent-samples t-test. When Levene’s test indicated unequal variances (p < 0.10), Welch’s t-test was used. For categorical variables, Fisher’s exact test was used throughout because of the small size of the moderate-to-high stigma group and the presence of sparse cell counts in several comparisons.

Effect sizes were reported with 95% confidence intervals. Cohen’s d, calculated using the pooled standard deviation, was used for continuous variables, with confidence intervals estimated using Hedges’ analytic formula. For 2 × 2 categorical comparisons, odds ratios (ORs) with 95% confidence intervals were reported, with the Haldane–Anscombe correction applied when a cell count was zero. Cramér’s V was reported for r × c comparisons and for place of residence, where the presence of a zero cell resulted in an unstable OR estimate; 95% confidence intervals for Cramér’s V were obtained using 5,000-resample nonparametric bootstrap procedures.

To account for multiple comparisons, raw p-values from the 18 bivariate tests were adjusted using the Benjamini–Hochberg false discovery rate (FDR) procedure. Both raw and FDR-adjusted p-values are reported. Substance use was not included in inferential testing because there was no variability in this variable. Given the small number of participants in the moderate-to-high stigma group (n = 13), the bivariate analyses were regarded as exploratory and hypothesis-generating rather than confirmatory. To specifically address potential confounding by age, an additional analysis of covariance (ANCOVA) was performed comparing ISMI total scores according to family history of death by suicide, with age entered as a covariate. The homogeneity of regression slopes assumption was assessed by testing the age-by-family-history interaction.

For the main analysis, multiple linear regression was used to examine factors associated with the severity of internalized stigma, with the ISMI total score entered as a continuous dependent variable. Variables were selected for inclusion based on findings from the preliminary bivariate analyses together with their clinical relevance as identified in the literature. Given the cross-sectional design, regression coefficients were interpreted as associations rather than causal or prospective effects.

To assess the robustness of the regression estimates and the potential instability associated with data-informed variable selection, a bootstrap sensitivity analysis was additionally performed. The regression model was re-estimated using 5,000 bootstrap resamples, and bias-corrected and accelerated (BCa) 95% confidence intervals were calculated for the unstandardized regression coefficients. To further assess potential overfitting and the out-of-sample performance of the regression model, repeated 5-fold cross-validation was performed as an internal validation procedure. The same prespecified set of predictors used in the final multiple linear regression model was retained throughout the cross-validation procedure. The data were randomly divided into five folds, with the model fitted on four folds and evaluated on the held-out fold. This procedure was repeated until each fold had served as the validation set, and the entire 5-fold cross-validation procedure was repeated 100 times using different random partitions. Out-of-fold predictions were combined within each repetition to calculate the cross-validated coefficient of determination (R²), root mean squared error (RMSE), and mean absolute error (MAE). The distribution of these performance estimates across the 100 repetitions was used to assess the stability of out-of-sample model performance.

Model assumptions were evaluated using standard diagnostic procedures. The distribution of residuals was assessed visually using a histogram and a normal P–P plot. Multicollinearity was evaluated using variance inflation factors (VIFs), and the independence of residuals was assessed using the Durbin–Watson statistic. All statistical tests were two-tailed, and p < 0.05 was considered statistically significant.

## 3 Results

### 3.1 Sociodemographic and clinical characteristics

A total of 97 individuals participated in the study. At the time of assessment, the mean score on the YMRS was 0.71 ± 1.56, and the mean score on the HDRS was 1.96 ± 2.29, confirming that the sample was in a stable euthymic phase.

The mean age was 41.07(± 12.43) years; 50.5% of the participants were female, and an equal proportion were married. Most of the participants lived in urban areas and mostly possessed a university degree or higher level of education.Approximately half of the participants reported smoking, alcohol use was minimal, and no participants reported illicit substance use.

A detailed summary of the participants’ sociodemographic and clinical characteristics is provided in **Table 1** and **Table 2**, respectively.

**Table 1:** Sociodemographic Characteristics of the Participants (N = 97).

Variable/Subcategory	Mean ± SD	
	N	%
**Age**	41.07 ± 12.43	
Gender		
Female	49	50.50
Male	48	49.50
Marital status		
Married	49	50.50
Currently single	48	49.50
Education (years)		
Primary school or below (≤8 years)	34	35.10
High school (≤12 years)	18	18.60
University (≥ 16 years)	45	46.4
Living arrangement		
Alone	3	3.10
With parents	32	33.00
With nuclear family	47	48.50
Other	15	15.50
Place of residence		
Rural	11	11.30
Urban	86	88.70
Current employment status		
Employed	39	40.20
Unemployed	39	40.20
Retired	15	15.50
Student	4	4.10
Smoking		
None	47	48.50
Present	50	51.50
Alcohol use		
Yes	11	11.30
No	86	88.70
Substance use		
Yes	0	0.00
No	97	100.00

N: Number of participants; SD: Standard Deviation. Categorical variables are presented as percentages.Values are shown as mean ± standard deviation or N (%).

**Table 2:** Clinical Characteristics of the Participants (N = 97).

Variable/Subcategory	N	%
Family history of any psychiatric disorder		
None	64	66.00
Present	33	34.00
Family history of death by suicide		
None	77	79.40
Present	20	20.60
Suicide attempt		
None	76	78.40
Present	21	21.60
	**Mean ± SD**	**Min–Max**
**Duration of illness (years)**	15.98 ± 11.29	0–50
**Age of BD onset (years)**	24.94 ± 9.05	14–55
**Number of hospitalizations**	2.58 ± 3.21	0–14
**Total number of episodes**	5.47 ± 3.86	1–21
Mania	3.64 ± 2.97	1–14
Depression	3.13 ± 3.30	1–20
Mixed	1.36 ± 0.74	1–3
ISMI		
Total score	55.68 ± 16.21	29–106
Mean score	1.93 ± 0.53	1–3.65
**RSES**	0.80 ± 0.85	0–5.42
**RSA – Total score**	126.64 ± 22.41	45–165

Clinical variables refer to lifetime history unless otherwise specified. N: Number of participants; SD: Standard Deviation. Categorical variables are presented as percentages(%).Values are shown as mean ± standard deviation or N (%).

ISMI: Internalized Stigma of Mental Illness Scale; RSES: Rosenberg Self-Esteem Scale; RSA: Resilience Scale for Adults.

Clinical characteristics of the participants, showed that those enrolled have a noteworthy background of illness and family-related risk (**Table 2**). About one-third of the sample (34.0%) had a family history of psychiatric disorders. Nearly one-fifth had a family history of death by suicide (20.6%) or personal history of suicide attempts (21.6%).

The illness course was chronic, with a mean illness duration of 15.98 years. Furthermore, the mean age of onset stood at 24.94 years. Participants showed an overall mean of 5.47 total mood episodes and 2.58 hospitalizations. Among the episode subtypes quantified separately, manic episodes had the highest mean count.

The average mean total score on the ISMI for the primary psychological measures was 55.68, which indicates low to moderate internalized stigma. The overall self-esteem of the study sample was high and the mean score of RSES was 0.80. The average total score of the RSA, which was 126.64, indicates moderate psychological resilience.

### 3.2 Preliminary analyses

In the analysis, participants were categorized into 2 groups based on their mean scores on the ISMI Scale (**Table 3**). Those participants who scored 2.5 and above were assigned to the “moderate to high stigma” group (n:13; 13.4% of the sample) while the rest 84 participants formed the “low stigma” group (n:84; %86.6).

**Table 3:** Comparison of Sociodemographic and Clinical Characteristics Between Low and moderate-to-high Stigma Groups.

Variable/subcategory	Low stigma (n = 84)	High stigma (n = 13)	Raw p	Adj. p (FDR)	Effect size (95% CI)
	Mean ± SD	Mean ± SD			
Age (years)	39.67 ± 11.97	50.08 ± 11.99	0.004	0.016*	- 0. 87^a^ (-1.47, -0.27)
	n(%)	n(%)			
Gender			1.000	1.000	0.86^c^ (0.27, 2.77)
Female	42 (50.00)	7 (53.80)			
Male	42 (50.00)	6 (46.20)			
Marital status			0.553	0.664	1.68^c^ (0.51, 5.55)
Single	43 (51.20)	5 (38.50)			
Married	41 (48.80)	8 (61.50)			
Education			0.330	0.533	0.16^b^ (0.04, 0.37)
Primary or below	27 (32.10)	7 (53.80)			
High school	16 (19.00)	2 (15.40)			
University	41 (48.80)	4 (30.80)			
Living arrangement			0.077	0.199	0.25^b^ (0.13, 0.45)
Alone	3 (3.60)	0 (0.00)			
With parents	31 (36.90)	1 (7.70)			
With nuclear family	39 (46.40)	8 (61.50)			
Other	11 (13.10)	4 (30.80)			
Place of residence			0.350	0.533	0.14^b^ (0.08, 0.20)
Rural	11 (13.10)	0 (0.00)			
Urban	73 (86.90)	13 (100.00)			
Employment status			0.884	0.994	0.11^b^ (0.07, 0.34)
Employed	34 (40.50)	5 (38.50)			
Unemployed	34 (40.50)	5 (38.50)			
Re tire d	12 (14.30)	3 (23.10)			
Student	4 (4.80)	0 (0.00)			
Smoking			0.236	0.497	2.36^c^ (0.67, 8.26)
Yes	41 (48.80)	9 (69.20)			
No	43 (51.20)	4 (30.80)			
Alcohol use			1.000	1.000	0.62^a^ (0.07, 5.26)
Yes	10 (11.90)	1 (7.70)			
No	74 (88.10)	12 (92.30)			
Substance use			—	—	—
Yes	0 (0.00)	0 (0.00)			
No	84 (100.00)	13 (100.00)			
Family history of any psychiatric disorder			0.356	0.533	1.81^a^ (0.55, 5.90)
None	57 (67.90)	7 (53.80)			
Present	27 (32.10)	6 (46.20)			
Family history of death by suicide			<0.001	0.005*	9.60^a^ (2.69, 34.31)
None	72 (85.70)	5 (38.50)			
Present	12 (14.30)	8 (61.50)			
Suicide attempt			0.470	0.604	1.75^a^ (0.48, 6.38)
None	67 (79.80)	9 (69.20)			
Present	17 (20.20)	4 (30.80)			
Duration of illness (years)	14.32 ± 9.98	26.69 ± 13.69	0.007†	0.022*	-1.18^a^ (-1.78, -0.57)
Age of BD onset (years)	25.23 ± 8.99	23.08 ± 9.55	0.428	0.593	0.24^a^ (-0.35, 0.82)
Number of hospitalizations	2.43 ± 3.09	3.54 ± 3.95	0.249	0.497	-0.35^a^ (-0.93, 0.24)
Total number of episodes	5.01 ± 3.65	8.38 ± 4.03	0.003	0.011*	-0.92^a^ (-1.52, -0.33)
RSA – Total	131.24 ± 18.27	96.92 ± 24.57	<0.001	<0.001*	1.79^a^ (1.15, 2.43)
RSES	0.61 ± 0.57	2.02 ± 1.28	0.002†	0.010*	-2.02^a^ (-2.67, -1.37)

Data are presented as Mean ± SD for continuous variables and n (%) for categorical variables. ^a^ Cohen’s d (pooled SD; 95% CI via Hedges’ analytic formula). ^b^ Cramér’s V (95% CI via 5,000-resample nonparametric bootstrap). ^c^ Odds Ratio (95% CI, Haldane-Anscombe corrected where a cell = 0). For continuous variables, Student’s t-test was used unless Levene’s test indicated unequal variances (p <.10), in which case Welch’s t-test (†) was used (Duration of illness, RSES). For categorical variables, Fisher’s exact test was used throughout. Cramér’s V is reported instead of OR for r×c comparisons (Education, Living arrangement, Employment status) and for Place of residence, where a zero cell makes the OR unstable.

“Adj. p (FDR)” is the Benjamini-Hochberg false discovery rate–adjusted p-value across the 18 bivariate tests performed. *p <.05 after FDR correction. Substance use was not tested due to zero variance. Higher RSES scores indicate lower self-esteem in the scoring system used in this study. Given the small size of the high-stigma group (n = 13), all bivariate results — including those surviving correction — should be interpreted as exploratory rather than confirmatory.

RSES: Rosenberg Self-Esteem Scale; RSA: Resilience Scale for Adults; OR: Odds Ratio; FDR: false discovery rate. * indicates p < 0.05.

Significant differences emerged between the two groups across several key clinical and psychological variables, and remained significant after correction for multiple comparisons using the Benjamini-Hochberg false discovery rate (FDR) procedure. Compared to those with low stigma, participants with moderate-to-high stigma were significantly older (mean age 50.08 vs 39.67 years, p = 0.004, FDR-adjusted p = 0.016), had a longer duration of illness (26.69 vs 14.32 years, p = 0.007, FDR-adjusted p = 0.022), and had a greater number of mood episodes (8.38 vs 4.96, p = 0.003, FDR-adjusted p = 0.011). A family history of suicide was also significantly more common in the moderate-to-high stigma group (61.5% vs 14.3%, p < 0.001, FDR-adjusted p = 0.005). The most difference was observed in the psychological measures. Individuals in the moderate-to-high stigma group had significantly lower resilience scores than those in the low-stigma group (96.92 vs 131.24, p < 0.001, FDR-adjusted p < 0.001). They also had significantly higher RSES scores (2.02 vs 0.61, p = 0.002, FDR-adjusted p = 0.010), indicating lower self-esteem in the scoring system used in this study. However, the groups did not differ significantly on any of the sociodemographic variables including gender, marital status, education and employment.

### 3.3 Advanced analyses

To identify the key factors associated with internalized stigma, a multiple linear regression analysis was conducted with the ISMI total score as the dependent variable. Given the cross-sectional design, the variables in this model were interpreted as statistical predictors/associated factors rather than causal or prospective predictors. In addition to the factors that were found to be significantly associated with internalized stigma in the bivariate analyses, clinically relevant other factors identified in the literature—such as gender, marital status, education, and place of residence—were also included in the model.

The overall regression model was statistically significant, F (10, 85) = 9.206, p < 0.001, and explained 52.0% of the variance in internalized stigma scores (R² = 0.520). Repeated 5-fold cross-validation (100 repetitions) indicated some attenuation in model performance compared with the apparent fit. While the regression model explained 52.0% of the variance in the original sample (R² = 0.520), the mean cross-validated R² was 0.372 (median = 0.369; 2.5th–97.5th percentile, 0.301–0.435). The mean cross-validated RMSE and MAE were 12.66 and 9.34, respectively. Thus, although the apparent R² was somewhat optimistic, a substantial proportion of the model’s explanatory performance was retained during internal cross-validation.

As detailed in **Table 4**, psychological resilience showed the strongest independent association with internalized stigma (β = –0.392, p < 0.001), with higher resilience associated with lower stigma levels. A family history of death by suicide (β = 0.203, p = 0.019) and higher educational attainment (β = –0.185, p = 0.029) were also significantly associated with internalized stigma. The total number of mood episodes showed a borderline positive association with stigma severity (β = 0.180, p = 0.050). Age, duration of illness, self-esteem, gender, marital status, and place of residence were not significantly associated with internalized stigma in the final model (**Table 4**). Because age was included in the multivariable model together with illness duration, number of mood episodes, and the other covariates, the reported regression coefficients represent associations adjusted for age and the remaining variables in the model.

**Table 4:** Multiple Linear Regression Analysis of Factors Associated with Internalized Stigma Severity (ISMI Total Score).

Variable	B (unstandardized)	Std. Error	β (standardized)	t	p-value	VIF
Family history of death by suicide	8.003	3.359	0.203	2.382	0.019*	1.290
Duration of illness	–0.219	0.173	–0.155	–1.267	0.209	2.639
RSA	–0.284	0.070	–0.392	–4.085	<0.001**	1.634
RSES	1.937	1.850	0.103	1.047	0.298	1.697
Number of episodes	0.751	0.377	0.180	1.991	0.050	1.452
Age	0.287	0.146	0.223	1.958	0.053	2.294
Education	–3.302	1.483	–0.185	–2.227	0.029*	1.222
Gender	–3.262	2.523	–0.102	–1.293	0.200	1.104
Marital status	1.741	2.573	0.054	0.677	0.501	1.147
Place of residence	1.989	3.886	0.040	0.512	0.610	1.062

B = Unstandardized coefficient; β = Standardized coefficient; VIF = Variance Inflation Factor. Model summary: R² = 0.520; Adjusted R² = 0.463. ISMI = Internalized Stigma of Mental Illness Scale; RSA = Resilience Scale for Adults; RSES = Rosenberg Self-Esteem Scale.(Higher RSES scores indicate lower self-esteem in the Turkish scoring system used in this study.) *p < 0.05, **p < 0.001. Bold p-values indicate statistical significance (p < 0.05).

The associations between the variables included in the multivariable regression model and internalized stigma are additionally visualized in **Figure 1** using unstandardized regression coefficients and their 95% confidence intervals.

**Figure 1:**
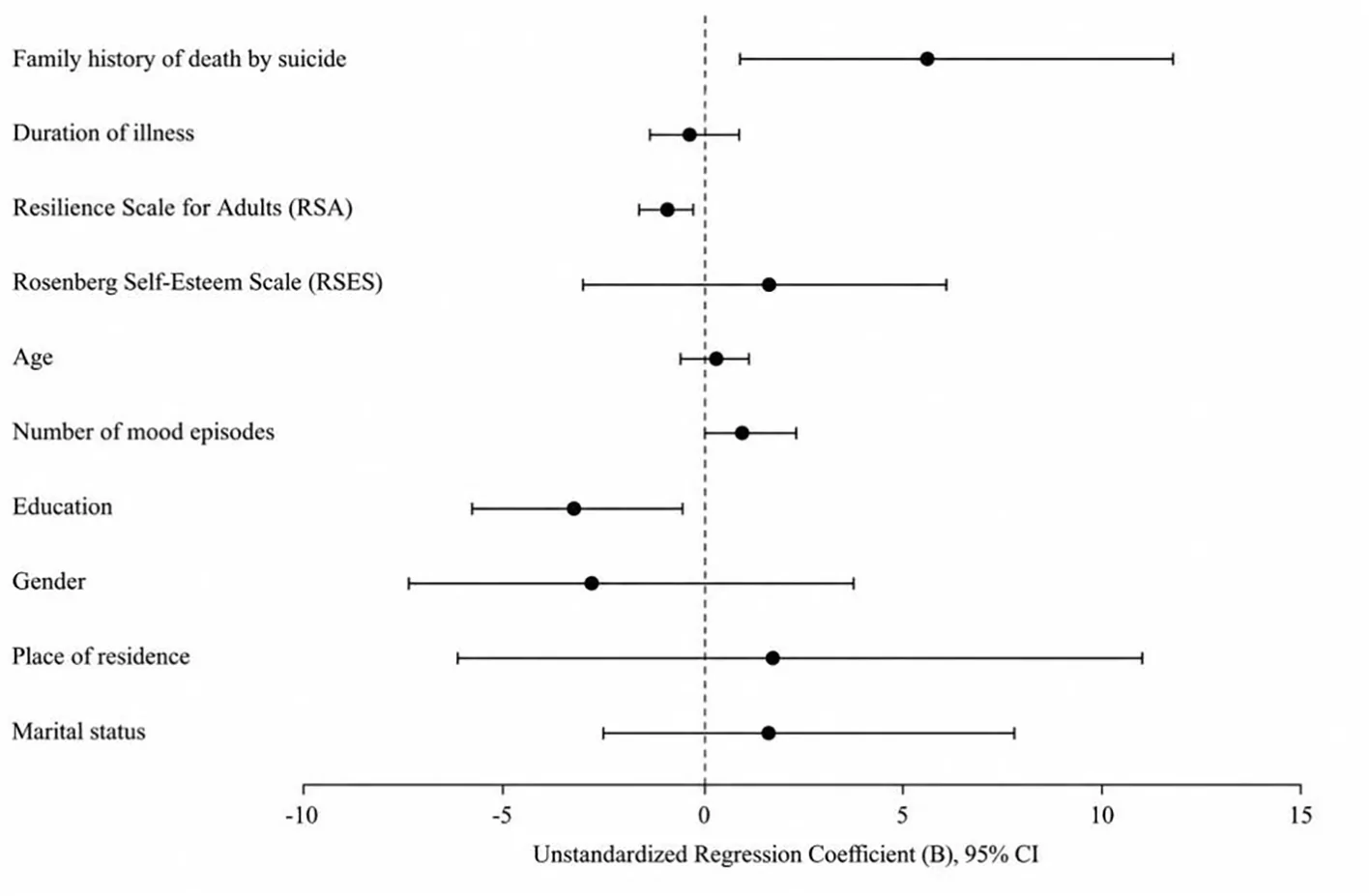
Forest plot of factors associated with internalized stigma in the multivariable linear regression model. Points represent unstandardized regression coefficients **(B)**, and horizontal lines indicate 95% confidence intervals. The dashed vertical line at zero represents no association. RSA, Resilience Scale for Adults; RSES, Rosenberg Self-Esteem Scale.

As a sensitivity analysis, the regression model was re-evaluated using 5,000 bootstrap resamples with bias-corrected and accelerated (BCa) 95% confidence intervals. Psychological resilience showed the most robust inverse association with internalized stigma (B = −0.284, BCa 95% CI −0.426 to −0.171). The number of mood episodes also showed a positive association (B = 0.751, BCa 95% CI 0.016 to 1.618). Age, which was borderline in the conventional regression model, showed a positive association in the bootstrap analysis (B = 0.287, BCa 95% CI 0.076 to 0.465). The estimate for family history of death by suicide remained positive (B = 8.003, BCa 95% CI 0.171 to 17.030), although the wide confidence interval indicated considerable uncertainty in the magnitude of the association. In an additional age-adjusted ANCOVA, participants with a family history of death by suicide had significantly higher ISMI total scores than those without such a history (adjusted mean: 63.30, 95% CI 56.78–69.82 vs. 53.70, 95% CI 50.39–57.02; F(1,94) = 6.77, p = .011, partial η² = .067). The adjusted mean difference was 9.60 points (95% CI 2.28–16.92). The age-by-family-history interaction was not significant (p = .656), indicating no evidence of heterogeneity of regression slopes.

## 4 Discussion

This study examined internalized stigma in euthymic individuals with bipolar disorder, aiming to identify its individual, familial, and clinical correlates. A family history of death by suicide emerged as an independently associated factor in the primary multivariable analysis, representing an original contribution to the literature by specifically focusing on a variable not yet systematically evaluated in this population. Psychological resilience and higher educational attainment were also associated with lower stigma, whereas a greater number of past mood episodes showed a borderline positive association with internalized stigma. Bootstrap sensitivity analyses indicated that the association with resilience was particularly stable, whereas estimates for family history of death by suicide and education were less precise and should be interpreted cautiously.

The findings build upon our initial bivariate analyses that showed that individuals with high internalized stigma were also older, had a longer illness duration, and reported lower psychological resilience, lower self-esteem, and a family history of suicide. While our study did not find significant bivariate associations for sociodemographic factors such as gender, marital status, or education level, these variables were nevertheless retained in the regression model because they have been found to be significant in previous research (38–43).

In our study, the mean total score on ISMI was found to be 55.68 ± 16.21, with a mean item score of 1.93 ± 0.53. According to the commonly used cut-off score of 2.5 in the literature, 13.4% of the participants were classified as having a high level of internalized stigma. This rate indicates a relatively low level of stigma compared to general psychiatric samples reported in both Turkish and international literature.When examining studies conducted in Turkey, this difference is thought to stem from the characteristics of the study population. Studies reporting higher stigma scores were conducted in non-specialized clinical settings, such as general psychiatric outpatient clinics or Community Mental Health Centers which shows significant differences in terms of sociodemographic variables compared to our study population from specialized mood disorders units of tertiary care centers (15, 44, 45). For instance, those in the studies were less likely to graduate from university (18–20%), reside in urban areas (27–37%), and be employed (27–31%). Past research has consistently related lower education, rural residence, and unemployment to higher stigma (15, 38, 41, 46, 47).

Our results are consistent with local studies performed on individuals with mood disorders in similar specialized tertiary care centers (4, 47). In these studies, both the percentage of the individuals with moderate-to-high stigma score (13.5%–18.5%) and the mean ISMI scores are comparable to our results. The common characteristics of individuals followed in these centers are a high level of education, urban residence, and being under long-term, regular follow-up in a specialized clinic.The international literature exhibits a similar pattern, as well. While studies in general psychiatric clinics typically report ISMI total scores in the range of 53–73 (48–50), research reporting scores similar to or lower than ours has predominantly been conducted in specialized centers for conditions like bipolar disorder (2, 51–53).

The relatively low stigma levels observed in our sample may reflect both the characteristics of individuals followed in specialized tertiary-care settings and features of these services, such as regular follow-up and access to psychoeducation. However, because different care settings were not directly compared, a protective effect of specialized clinic follow-up on internalized stigma cannot be inferred from the present findings.

Participants in the moderate-to-high stigma group were significantly older than those in the low-stigma group in the preliminary bivariate analysis. In the conventional multivariable regression model, however, the association between age and internalized stigma was attenuated and narrowly missed the conventional threshold for statistical significance after adjustment for illness duration, number of mood episodes, and the other covariates. In contrast, the bootstrap sensitivity analysis supported a positive association with age. This discrepancy suggests that the estimated contribution of age may be sensitive to the analytic approach and sampling variability. Although age was moderately correlated with other indicators of cumulative illness burden, multicollinearity was not substantial. Accordingly, the potential independent association between older age and greater internalized stigma should not be regarded as definitively absent, but should be interpreted cautiously and replicated in larger samples.

The literature is also not entirely consistent regarding the relationship between age and stigma (13, 54). One possible explanation for the observed bivariate association is that age may partly reflect cumulative illness burden over time, given that bipolar disorder commonly begins in early adulthood and follows a chronic course (1). Repeated mood episodes, prolonged treatment experiences, and disruptions in social and occupational functioning may contribute over time to negative self-appraisals and internalized stigma. Therefore, the association observed with age may represent both an age-related effect and, at least partly, the accumulated consequences of illness chronicity.

Participants in the moderate–high stigma group also had a longer duration of illness in the preliminary bivariate analysis. However, illness duration was not independently associated with internalized stigma after adjustment for age, number of mood episodes, and the other covariates in the multivariable model. This suggests that the bivariate association with illness duration may partly reflect its close relationship with age and cumulative episode burden. Nevertheless, previous studies that showed a positive correlation of the duration of illness with ISMI scores in severe mental disorders (46). As the condition progresses, individuals may accumulate experiences such as mood episodes, hospitalizations, and treatment changes. Although this accumulation may in some cases foster greater illness awareness, it may also contribute to a strengthened “patient identity,” potentially reinforcing internalized stigma over time.

A greater number of lifetime mood episodes showed a borderline positive association with internalized stigma in the multivariable model (β = 0.180, p = 0.050). The finding shows consistency with past research suggesting that frequent episodes are related to higher stigma (54–56). These findings support the notion that multiple episodes may encourage internalized stigma, while increased stigmatization may limit treatment adherence and result in new episodes (40). Studies focusing on episode frequency also support this relationship. Tanyeri Kayahan and Vahip (2025) (47) found that both the number of episodes and rapid cycling were associated with internalized stigma but were not identified as predictors. In contrast, Üstündağ and Kesebir (2013) (15) suggested that rapid cycling could be associated with higher stigma levels.

Among the clinical burden variables entered into the multivariable model, the total number of mood episodes retained a borderline positive association with internalized stigma after adjustment for age, illness duration, and the other covariates. Illness duration was not independently associated with stigma severity, whereas the association with age was borderline in the conventional regression model but was supported by the bootstrap sensitivity analysis.

In the primary multivariable model, higher educational attainment was negatively associated with internalized stigma. However, the bootstrap confidence interval approached the null value, indicating limited precision and warranting cautious interpretation of this association. The findings were consistent with previous studies indicating that individuals with lower levels of education reported higher levels of internalized stigma (15, 41, 46, 54, 57). Such a finding is clinically relevant since bipolar disorder usually occurs in young adulthood, which is a vital period for education and work-related activities (1). As such, efforts to help young people stay engaged with or return to school might be an effective long-term intervention to prevent the internalization of stigma and improve outcomes. Being educated may improve health knowledge and treatment compliance which will reduce hospital admission and the frequency of episodes.

Consistent with previous studies, psychological resilience showed a strong negative association with internalized stigma in our model and remained the most stable association in the bootstrap sensitivity analysis (2, 17).

Because resilience is linked to stigma resistance, self-esteem, and quality of life, it may represent a clinically relevant and potentially modifiable target for interventions aimed at reducing internalized stigma (56). These findings suggest that resilience may represent a valuable and potentially modifiable target for interventions aimed at reducing internalized stigma. Integrating resilience-building strategies into anti-stigma programs, such as those using psychoeducation and cognitive techniques, may therefore be a clinically useful approach (13, 55).

Consistent with a large body of literature, lower self-esteem was significantly associated with higher internalized stigma in our bivariate analysis (41, 58–60). After adjusting for other covariates in the multivariable regression—particularly the more robust construct of resilience—self-esteem was no longer a significant independent factor. This attenuation is likely due to the shared variance between self-esteem and resilience, with the latter accounting for the effect in the final model.

An important finding of our study was the association between a family history of death by suicide and higher internalized stigma in the primary multivariable model. However, the bootstrap confidence interval approached the null value, suggesting that the precision and stability of this estimate should be interpreted cautiously. There are not many studies that look for specific links in Bipolar Disorder. One recent investigation reported an initial association, but that effect did not continue through their multivariable analysis (59). On the other hand, Tanyeri Kayahan and Vahip (2025) (47) study lends full support to our findings. An individual with a family history of a suicide attempt or death by suicide scored significantly lower in stigma resistance section of ISMI. This finding suggests that a family history may make some individuals more vulnerable to internalized stigma, although this interpretation should be considered cautiously.In the literature at large on suicide bereavement, one finds the overwhelming power of stigma. Stigma is the most harmful social factor, with survivors reporting intense feelings of shame, guilt, and social isolation (61–63). A family history of suicide in BD is a marker of a severe clinical phenotype. This is evidenced by an earlier age of onset, more comorbidities as well as a higher personal risk of suicide attempts (64, 65). It could be hypothesized that this dual burden—a more severe clinical phenotype alongside the psychosocial weight of a family member’s death by suicide—may independently contribute to internalized stigma. Drawing on the broader suicide bereavement literature, one possible explanation is that feelings of shame, guilt, and social isolation experienced by surviving family members may become internalized over time, potentially amplifying stigma; however, these psychosocial mechanisms were not directly measured in the present study and therefore remain speculative. Similarly, it could be hypothesized that stigma-related shame associated with a family history of suicide may contribute to treatment avoidance and reduced help-seeking behavior. For individuals already vulnerable due to a more severe illness course, such avoidance could in turn be associated with more frequent episodes, thereby potentially reinforcing internalized stigma—though this proposed cycle requires prospective investigation to be substantiated. Accordingly, the proposed treatment-avoidance pathway should be interpreted as a hypothesis for future research rather than an empirically tested mechanism.These hypotheses represent important directions for future research and underscore the importance of systematically assessing for a family history of suicide in clinical practice.

From an intergenerational perspective, these findings may also reflect a cumulative burden extending beyond the direct clinical vulnerability associated with bipolar disorder. A family history of death by suicide may carry not only familial and clinical vulnerability but also the psychosocial consequences of suicide bereavement, including shame, blame, secrecy, and social judgment (19, 20). The coexistence of these familial and psychosocial influences may contribute to a cumulative stigma-related burden across generations. In this context, the concept of “intergenerational burden” may help describe the combined impact of familial suicide history on individuals with bipolar disorder. However, because these intergenerational processes were not directly assessed and the present study was cross-sectional, this interpretation should be regarded as conceptual rather than causal.

In conclusion, internalized stigma is a critical clinical issue that can adversely affect the course of bipolar disorder. Our findings suggest that a family history of death by suicide may be associated with higher internalized stigma, although this association should be interpreted cautiously in light of the bootstrap sensitivity analysis. A greater number of mood episodes also showed a borderline positive association with stigma severity. Higher educational attainment and psychological resilience were associated with lower internalized stigma. Therefore, interventions should move beyond symptom management to build psychological resilience—providing the dual benefit of reducing stigma while improving clinical outcomes—and to systematically assess for a family history of suicide. This assessment is important as it may identify individuals who are more vulnerable to higher internalized stigma, warranting additional psychosocial support. By addressing these factors, clinicians may make a meaningful contribution to reducing stigma and improving the long-term prognosis of individuals with bipolar disorder.

## 5 Limitations

This study has several limitations. First, its cross-sectional design precludes the establishment of causal relationships between variables. Second, since the sample was drawn from one specialized mood disorders care center in a single city, the generalizability of the findings may be limited. Third, the reliance on self-report for variables such as family history of suicide could introduce recall bias. Fourth, although the overall sample size was adequate based on the *a priori* power analysis, the modest sample size and the small number of participants in the moderate–high stigma group (n = 13) represent important limitations. Although age was included as a covariate in the multivariable model, residual confounding cannot be excluded, particularly as the bootstrap analysis — unlike the conventional model — suggested a significant independent association for age. The observed association between age and family history of death by suicide in the bivariate comparisons may partly reflect greater cumulative exposure time in older participants; however, this association persisted after adjusting for age in the multivariable model, suggesting that it is not solely attributable to age. In addition, although bootstrap resampling was used to assess the stability of the regression coefficients, the relatively modest sample size in relation to the number of covariates included in the model raises the possibility of overfitting. Therefore, the explained variance and individual coefficient estimates should be interpreted cautiously and require validation in independent samples. The bivariate comparisons between stigma groups should likewise be interpreted as exploratory and descriptive rather than definitive. The imbalance between the low- and moderate–high-stigma groups may have reduced statistical power for detecting some associations and may have increased the instability of estimates for variables with sparse categories. Fifth, the psychosocial mechanisms potentially linking a family history of suicide to internalized stigma—such as feelings of shame, guilt, or treatment avoidance—were not directly assessed using validated instruments. Consequently, the interpretations offered regarding these pathways in the Discussion are hypothetical in nature and should be regarded as directions for future research rather than conclusions supported by the present data. Sixth, the total number of mood episodes was assessed retrospectively through clinician interview and electronic medical record review. Given the mean illness duration of approximately 16 years, this approach is susceptible to recall bias, particularly for hypomanic or less severe depressive episodes that may not have resulted in formal clinical contact. Because hypomanic episodes could not be consistently verified from clinical records, they were not quantified as a separate episode-count variable. Therefore, episode counts should be interpreted as approximate indicators of lifetime illness burden rather than precise lifetime counts. Seventh, the sample was predominantly urban (88.7%) and drawn from a tertiary-care specialized clinic, with relatively high educational attainment; these characteristics may limit the generalizability of the findings to individuals with bipolar disorder in general psychiatric or community settings.

Eighth, internalized stigma was assessed exclusively during a sustained euthymic phase. Because ISMI items reflect participants’ current perceptions, feelings, and stigma-related concerns, stigma severity measured during euthymia may underestimate the burden experienced during symptomatic periods, particularly depressive episodes. In addition, participants were recruited from individuals who were clinically stable and regularly attending follow-up appointments at a specialized mood disorders clinic. This may have selected for individuals with better treatment engagement, greater clinical stability, and more favorable psychosocial outcomes, thereby introducing selection or survivor bias. Consequently, both the prevalence and severity of internalized stigma observed in this sample may be lower than those in less stable, untreated, or acutely symptomatic populations.

The use of the Turkish Guttman-scored version of the RSES also limits direct comparability with studies using the more commonly applied Likert-summed scoring system. Although the direction of scoring was explicitly considered in all analyses, differences in scoring methodology should be taken into account when comparing self-esteem findings across studies.

Finally, item-level responses for the psychometric instruments were not available in the final analytical dataset, precluding calculation of Cronbach’s alpha coefficients for the present sample. Therefore, the internal consistency of the ISMI, RSA, and RSES could not be directly evaluated in this specific study population, and interpretation relies on the previously established reliability of the validated Turkish versions of these instruments.

## 6 Clinical implications

There are several clinically relevant implications of this study’s findings. In the regression model, family history of death by suicide was significantly associated with internalized stigma (β = 0.203, p = 0.019), while the total number of mood episodes showed a borderline association (β = 0.180, p = 0.050). These findings suggest that familial context should be considered in stigma-sensitive clinical assessment, while the possible contribution of cumulative illness burden warrants further investigation in larger longitudinal studies. Although a family history of death by suicide is not itself a modifiable factor, it may help clinicians identify a subgroup of individuals who could benefit from closer follow-up, suicide-focused psychoeducation, and additional psychosocial support addressing stigma-related vulnerability. Similarly, the total number of mood episodes may be considered a marker of cumulative illness burden, highlighting the importance of sustained mood stabilization and relapse prevention as part of stigma-sensitive clinical care. More broadly, future family-based longitudinal studies should examine whether suicide prevention in bipolar disorder has implications beyond individual with bipolar disorder, including a possible reduction in the psychosocial and stigma-related burden experienced by family members.

Psychological resilience showed the strongest negative association with internalized stigma in the regression model (β = –0.392, p < 0.001), suggesting that resilience-building components may be useful in anti-stigma interventions. Together with broader community mental health literacy and school-based anti-stigma education, the association between educational level and internalized stigma emphasizes the importance of long-term educational and awareness-based strategies.

In a collectivist culture like Türkiye, where stigma is often not expressed explicitly, anti-stigma strategies should be culturally sensitive.

## Data Availability

The raw data supporting the conclusions of this article will be made available by the authors, without undue reservation.
